# Exploring research capacity and capability in a local authority: qualitative insights from leaders and staff

**DOI:** 10.1186/s12889-025-23705-0

**Published:** 2025-07-15

**Authors:** James Woodall, Chloe Bracewell, Andrew Passey, Samantha Start, Jane South

**Affiliations:** 1 School of Health, Leeds Beckett University, Leeds, LS1 3HE UK; 2 Wakefield Metropolitan District Council, Wakefield, WF1 UK

**Keywords:** Research capacity, Local authority, Evidence-based policy, Health determinants, Qualitative insights, Organisational culture

## Abstract

**Background:**

Local authorities in England are ideally placed to address the social determinants of health in the communities they serve. An evidence-led approach to developing programmes and policies to tackle determinants of health is critical to ensuring outcomes are attained and resources are used appropriately. Previous studies though suggest that local authorities do not always use evidence consistently in their decision-making processes. This paper seeks therefore to explore perceived research capability and capacity across one local authority in northern England to understand how research influences policy and practice.

**Methods:**

A qualitative exploration of 29 leaders and managers across the local authority, representing the four directorates of the organisation, was obtained to gain an overall understanding of research capacity and capability. Data were analysed thematically with eight overarching thematic categories derived.

**Results:**

The capacity and capability for research across the local authority directorates varied. Some participants described departments within directorates as being research active where research was part of their core business. Conversely, some departments were engaged in front-line service delivery where research was not prioritised. In these areas there was a disconnect between daily working practices and research. Staff in these departments generally lacked skills and training in research, whereas those in research active areas often had professional training where research was incorporated. There was rarely a shared definition of research by participants and ambiguity in what constituted research was common. The local authority was perceived to gather lots of data, but this was often used very functionally to fulfil reporting obligations. Curiosity to explore data was often minimised due to work pressures. Links from local authority staff to democratically elected officials varied and research and evidence was not always routinely presented. The majority of participants recognised that reforming ways of working and developing a clear training offer around research would be beneficial to addressing health outcomes.

**Conclusions:**

Data demonstrated variance between research practice, partnerships and culture in departments where space for intellectual curiosity was tempered by service demands. There were exceptions to this, where departmental views of research were positive and leaders valued the research-informed culture.

## Background

Local authorities in England are the elected municipal bodies that serve specific geographical areas and have responsibility for the delivery of a range of public services [1]. Local authorities are frequently described as the tier of government closest to the community as they operate within metropolitan and regional areas of a state [2]. In England there are 317 local authorities that are well-placed to bring about improvements in health and well-being for the citizens they serve through ‘bottom-up’ engagement and ‘top-down’ policy directives [2, 3]. Indeed, there is a growing acceptance that health aligns to socioeconomic status [4] and that factors under the control of local authorities (such as the quality of housing; education; and employment) have a key role in determining health outcomes [5]. Local authorities therefore have a pivotal role in addressing the social determinants of health and their contribution to reducing health inequalities is now unrefuted [6]. Local government has legal obligations to improve population-level health [7], and is also highly influential in other policy and practice areas, including housing, education, employment and skills, parks and gardens, and transport.

Although contested by some [8], an evidence-led approach to developing policy and practice in local authorities to tackling health inequalities is seen as highly preferential for ensuring that finite resources are used carefully and with a high likelihood of being effective [1]. Given funding constraints and current drives for efficiencies in how local authorities operate, evidence is also useful in shaping internal organisational change [9]. The notion of what constitutes ‘evidence’ though within local authorities is not agreed and indeed highly variable [1, 10]– it can, however, include a spectrum of information, such as academic research, local administrative data, information from community forums and experiential knowledge. Despite the promise of addressing health inequalities in communities and addressing population health, there are many issues that prevent evidence-based policy from being enacted. While most people working in local government recognise the importance of research and indeed use research in their practice [10], capacity and skills are frequently cited as barriers to utilising research to inform policy-making. The tension that staff in local authorities may experience between the pressure to produce recommendations quickly for political reasons and the often time-consuming process of using rigorous research data and evidence in policy development has also been noted [3]. Moreover, the nature of siloed working with local authorities and blockages in evidence-flow between departments with a clear impact on health determinants can be common [1]. Solutions have been proposed to embed a stronger research culture in local authorities, including the deployment of Embedded Researchers who straddle academia and local government [11]; and directly contracting more research staff in local authority areas to improve evidence generation and analysis [12].

Differences in research capability and capacity are well-explored in healthcare systems, particularly the NHS [13, 14], but less focus has been on local authorities. To support the development of evidence-informed decision-making across all areas of local government and ensure that research is conducted where social and public health needs are greatest, it is essential to build research capacity across all departments and for all staff groups. West and colleagues [10] developed a typology relating to research activity in local authorities, identifying four ‘types’ of research culture and environment ranging from negligible activity (type 1) to comprehensive and systemic approaches (type 4). Their research focusing on Bradford Metropolitan District Council in the UK, suggested activity was overall at type 2. Further research has identified six types of research ‘systems’ operating within local authorities [12]. A critique of these methodological approaches is that assessing research capability across the entire local authority can overlook the differences in smaller departments. Indeed, some departmental areas use research as part of their daily practice and have opportunities to develop research skills [15] whereas other departmental areas cite anxiety and apprehension when considering research as part of their practice [16]. As an example, the shift of public health responsibility moving from health authorities to local authorities saw *“an injection of evidence-based models into the local decision-making arena”* ([12], p.2), this paper seeks to achieve this through sampling a wide range of stakeholders in one local authority capturing the diversity of research engagement within local authorities– providing insights that potentially go beyond broad organisational typologies [10].

Within the United Kingdom, the Health Determinants Research Collaboration (HDRC) is a programme which seeks to increase research capacity and capability within local government to understand health determinants and to improve health outcomes in communities [17]. This is not the first national attempt to address inequalities in the UK, with several policy initiatives and delivery mechanisms established to lessen poor health for communities [18]. The HDRC programme supports thirty local authorities who work with academic partners; voluntary and community sector organisations; decision-makers; and citizens to better understand health influences and to create conditions that will lead to improved health outcomes [19].

As part of understanding the current research landscape of one local authority that has recently been awarded HDRC status, a qualitative approach was taken to explore current research capability and capacity across the organisation. The variety of research and evidence relevant in a local government context was recognised and no predefined view of what constitutes ‘research’ or ‘evidence’ was given or implied in the study. Epidemiological data in this local authority suggests high levels of health inequality and lower than national average life expectancy. The authority, which is situated in northern England, covers over 300 square kilometres, covering both urban and rural communities. It is described as a ‘metropolitan district’ council, meaning it has responsibility for all local government services within its area, including: education, transport, planning, social services and waste management [20]. A purposive sample of leaders and managers were identified to give insight into research capability and capacity across all departments of the organisation. The aim of the research was to gather a more subtle understanding across local authority departments to inform future staff development and support around research and evidence to inform evidence-based policy programmes.

## Methodology

While some studies have adopted quantitative surveys to ascertain research capability and capacity and show the breadth of activity across a local authority [10], a qualitative approach offered the opportunity to understand the differences within the local authority. Such understanding is currently lacking in the extant literature. A maximum-variation sample was adopted to cover the broadest range of information and perspectives in the authority at managerial and leadership levels [21]. This resulted in the four main directorates (Regeneration, Environment & Economic Growth; Adults, Health & Communities; Children & Young People; and Resources), and a myriad of departments within each of these directorates, included in the sampling frame. The sampling sought to focus on key informants within local authority departments, particularly service managers and directors. These key informants were often responsible for leading teams and service areas and could provide broad perspectives both within the department and comment more widely, because of their strategic role, on wider perspectives outside of their immediate areas in the local authority. Individuals were contacted via email to participate and 29 participants were included in data collection, representing the four main directorates within the authority.

Recognising previous experiences of data gathering with local authority leaders and staff [3], the team were mindful that pragmatism in data collection approaches would be crucial to the likelihood of success. In response to this, individual semi-structured interviews and focus group discussions were offered as an opportunity to optimise data gathering. Seven individual interviews, two dyadic interviews and three focus groups ranging from five to seven individuals per focus group took place with the participants. Focus groups with participants from the Resources directorate and the Regeneration, Environment & Economic Growth directorate, as well as dyadic interviews with participants from the Children & Young People directorate, were conducted at the request of the teams. This was because data collection was often scheduled to coincide with existing team meetings. Acknowledging that both semi-structured interviews and focus groups have their unique strengths, it also enabled greater flexibility for local authority colleagues and potentially increased response rates to the study. The qualitative discussion guide was adapted from a prior study the lead author had been involved with [7] as well as a review of the current evidence base. The guide focussed, therefore, on understanding current research skills and expertise in teams; barriers to research use and activity; links with external research organisations; how evidence and data are derived and disseminated; and how evidence informs decision-making. All aspects of the study were approved by Leeds Beckett University ethics committee.

Qualitative data gathering was done via MS Teams and a transcript produced from these discussions. This transcript was later ‘cleaned’ to ensure data quality and accuracy through listening to the qualitative data and comparing this with the transcription. Three of the authors (JW, CB, AP) independently coded a selection of transcripts and came together to agree a coding framework that could be applied consistently to the dataset. There were minor discrepancies in the codes, but any inconsistencies were resolved by discussion. Focus group data were analysed separately noting any inconsistencies in participants’ views; however, a coding framework, developed through familiarisation and analysis of all of the data gathered, enabled a replicable approach to be used by multiple analysts. This framework consisted of eleven codes, developed through both inductive and deductive processes, which were then applied to the data. Nonetheless where additional codes were required to exemplify an important aspect of the data, an iterative approach allowed the coding framework to be expanded. Eight higher-order themes (presented in the next section) were derived through grouping codes and aggregating similar salient issues arising in the data.

## Results

This section presents themes deriving from the process of data analysis. Eight thematic categories are presented in this section, with quotations used to exemplify and illustrate issues discussed. We have not attributed the quotations due to the potential to compromise anonymity. The participant sample achieved maximum variation of role and covered all four directorates in the local authority.

### Heterogeneity in research culture

Across the data set, there was clear differences in research understanding and confidence and the ethos and culture regarding research in departmental areas. In a minority of departments, research was seen as a regular part of working routines and staff in these areas felt equipped to gather, analyse and interpret research to aid their professional practice. Engagement with contemporary research and research techniques and methodologies were reported by participants to be embedded in the culture of the departments and supported by leadership in these areas. Participants working in public health and in transport and planning were described as having a strong research and evidence ethos in the development of programmes and interventions. Prior training and disciplinary expectations and norms were often cited as mechanisms increasing research engagement:

There are one or two directorates where research is probably reasonably well used in terms of that there’s an evidence based to the way that work is carried out.

In other departments, conversely, staff suggested that their teams lacked confidence, training and skills in research. Some participants even suggested that research was intimidating and held negative perceptions about the process for research. Engagement with research was less evident, particularly in activities such as searching and evaluating literature or analysing and interpreting data. These were often viewed as separate from, and less integrated into, day-to-day work responsibilities.

Development opportunities were also suggested to lack consistency across the authority. Some participants reflected on robust pathways for professional training and the important influence of regulatory bodies (e.g. Faculty of Public Health) in developing research competency, but this was not apparent in all disciplinary areas. Internal opportunities were also inconsistent, with no clear route for staff to develop research skills. Continuous Professional Development (CPD) related to research was typically driven by individual managers in one-to-one discussions and performance reviews rather than a structured, organisation-wide approach. Leaders who themselves had an interest in research seemed more likely to encourage others in their time to develop research skills through training.

### Ambiguity and varied terminology surrounding ‘research’

The discourse concerning ‘research’ did cause some confusion and misgivings from participants, with clarification often sought regarding the definition constituting ‘research’:

So can I just clarify that research, to me, is data collection…I don’t want to get the definition wrong…because I’m not sure that research is a word that we use in my area.

Some participants reported that the term ‘research’ was commonly understood and recognised by team members. However, in other departments terms such as: ‘evidence’, ‘evaluation’, ‘intelligence’ and ‘reflection’ were deployed. In social care, for example, experiential learning was seen to be a critical form of evidence for improving practice. Delineating the nuances between these concepts was difficult for participants and moreover often terms were used interchangeably during discussions:

It’s whether we use that term research, but we use data all the time in terms of decision making.

It was clear that a shared definition of what constituted ‘research’ was not routinely applied either across the local authority, or within departments. Instead, more implicit and tacit understandings seemed to be used when discussing how research was used in practice:

I think that there are parts of my service that probably do it [research] without knowing they’re doing it.

On balance, it was apparent that the local authority was more versed in consuming research and evidence than producing or disseminating research from their own data gathering or analytical activities. Most participants recognised how to access information sources to inform their planning and programmes, but evidence generated by the local authority was less frequently disseminated in formal ways. Rarely did interviewees discuss disseminating their own findings in academic journals as there was very little motivation to do so. Nonetheless, sharing local information across professional networks was not uncommon.

### External links and partnerships

There were inconsistencies in the strength of academic and external partnerships across different departments. The analysis demonstrated departments with strong links with universities and external bodies– this could include relationships with academics to support evaluation activity or departments being involved themselves in research studies (to support research recruitment, for example); or hosting PhD students in one case. The local authority did not have a university in its direct geographical region, but participants perceived that, in theory, this offered good opportunities to maximise relationships with a range of organisations in the wider locality. In reality though, some departments had less robust arrangements and limited external partnerships beyond a very small network. Engagement with universities, as an example, was less apparent with any established connections based on opportunistic arrangements or more precarious foundations:

Working along with the university would be something we’d really like. You know, we’ve always sort of kind of looking to maybe try and do that, but we’ve never quite been able to sort of make it happen at the right time.

### Disconnect between research and daily work

In the majority departments within the directorates, the pressures and demands of the respective areas meant that research was often seen as a luxury or done ‘in addition’ to the daily requirements of the role. Participants emphasised the demands on their services’ time and resource and had no additional capacity to engage in research, even though many saw the value in an evidence-led approach to policy and programme development. There was a tendency to prioritise statutory obligations over research, leading to limited engagement with research activities which were often seen as secondary.

Some departments were described as being ‘highly regulated’– finance and legal services, for example– and process driven which stifled any activity perceived to be ancillary to the main business. Yet some leaders were frustrated by this and wanted a more proactive approach to doing things differently, even to benefit internal processes and structures within the authority. Some examples of radical innovation were provided in directorates, where research and evidence had been used to re-design fundamental ways of working. This, however, was often due to under performance or a regulatory breach which made change processes essential and easier to implement.

Time for intellectual curiosity in the working day was often limited and opportunities to explore problems or read research studies were uncommon. As staff resource was stretched across busy directorates, opportunities to undertake proactive research was not a possibility even if enthusiasm by colleagues was present:

[Staff] are out there doing the business, they’ve not got the time to sit down and write and publish and that kind of thing.

In a minority of departments with directorates though, as mentioned, research and daily practice were completely intertwined and inseparable, providing a strong research culture and a recognition of its importance in delivering high-quality services. Those working in planning within the local authority articulated the fundamental use of a range of research for policy and development:

Every policy we have has some sort of research behind it, whether it’s primary research or whether it’s secondary research. Looking at studies and other things. We also rely on public consultation, which is a form of research.

Participants perceived that the public health department within the local authority was also an area where research and engagement with evidence was embedded in working practices. This was apparent in developing programmes and interventions, but also more subtly in relation to staff finding time to read the latest research relating to their roles and responsibilities.

### Siloed working

Working holistically and across directorates within a local authority was not always described as being comfortable or easy. There were natural affiliations between areas of the local authority that had synergistic benefits, but mostly inter-departmental work was often too challenging and resource intensive. Research and evaluation activities were often conducted in silos, with limited sharing of findings across teams. This results in a lack of coherence and missed opportunities for cross-departmental learning and improvement:

If I had a magic wand, it would be that we were able to remove some of the barriers to sort of innovate and research different approaches to doing things, but across a range of sectors where they interconnect,

This challenge was further compounded by funding structures available to the local authority, which often reinforced siloed ways of working. These funding opportunities from national grant bodies (NIHR, as an example which was given by some participants) frequently mis-aligned with the complex, long-term research needs of the organisation. This was reported to contribute to the challenges in sustaining meaningful research initiatives and in collaborating across departments.

### Functional data application and usage

Data were perceived to be frequently used for performance management and compliance with statutory obligations, rather than for innovative or community-specific initiatives in many of the directorates. Often directorates were rich-data environments, but typically this information was used in responding to statutory obligations or reactive needs rather than for answering specific research questions or challenges that could benefit local people:

There could be more done with the knowledge and data we have in order to inform what we do and improve our outcomes for citizens.

Time constraints, again, were a contributory mechanism to the limitations in data application. The data-rich environment in many directorates was clearly beneficial for evidence-based decision-making, but there was an appreciation that the data were not being maximised. In some cases, specialist consultants were commissioned to undertake analysis or similar activities to yield more insight from existing data sources.

### Working with elected members

Given the seniority of participants interviewed, they were highly-cognisant of the politicised nature of local government and indeed were acutely aware of the systems and democratic processes within the organisation. However, the connections between departments, research-evidence and elected members within the local authority varied. Some participants recognised the challenges and difficulties of the elected members role but highlighted how research evidence may not be sufficiently ‘localised’, or that more often members were *“led by their gut and who’s loudest in their constituency”*. Some respondents described having regular meetings with named elected officials and had developed communication channels to inform and communicate to enable effective decision-making:

It is about keeping Members informed and more often than not, it’s about explaining why we can’t do something more than we can do something.

In contrast, other departments had more informal arrangements or seemed detached from the political nature of the organisation. In some departments, the elected member was not always at the centre of discussions or necessarily engaged or trained to understand research evidence to enact change. One participant suggested more training for elected officials:

I think that’s how we help Elected Members develop, I think is a big part of where the Council needs to go because we’ve talked a lot about offices and developing officers, but not Members.

### Organisational change required

There was a universal view that organisational change– including culture, structures and processes– was necessary in order to create widespread changes within the local authority in relation to research. To create a larger cultural shift, there were several strategic and operational changes that needed be implemented to embed research in the organisation. Creating space and time for colleagues in the role was perceived as critical in shaping a more research-informed approach as well as de-mystifying research and creating a common understanding across council staff and elected members:

Obviously statutory work is always going to take precedence over some of this stuff. But I think if there was like a bit of a culture created around it over time that would help to really create space for this.

I think we need to demystify it. And maybe change the lexicon and then we can move forward from there really. Both on the officer side and members…I think that’s the way we probably need to develop that that conversation. It’s not just about this scary word.

Participants also wanted a stronger leadership approach to research where there was ‘permission to play’ and be intellectually curious. In addition, senior leaders in the organisation modelling and being involved in research activity was considered an important way to consolidate and grow a research culture:

I think if there was a bit more of a top-down approach…it’s just really making it explicit that this is allowed and there’s permission for this kind of development. I think that would really help…allowing it, but also modelling it.

## Discussion

This paper sought to understand and explore research capability and capacity within a local authority, given that evidence-based decision-making can provide a better chance of policy being made that impacts positively on populations and communities [1]. A more nuanced approach to the topic was employed following calls in the international literature to have greater understanding of evidence use and generation in local authorities [2]. A purposive sample was used to provide understanding across all four directorates of the organisation to enable a detailed view of the organisation. The analysis showed that participants perceived both commonality across the organisation and significant variability in capability and capacity in departments within the directorates– these issues will be discussed further below.

The constraints on time for many staff was a common factor which inhibited the ability and inclination of the workforce to engage in research activity. This sentiment is common in the extant literature and has been identified previously [7]. In many departments of the local authority there was a disconnect between research *and* daily work. Research was seen as something that sat outside of, and distinct from, statutory duties and obligations. Participants perceived that there was an appetite for research with departments but, like other studies [22], a feeling that daily duties and citizen demand overwhelmed and inhibited research engagement. There were some exceptions to this and some departmental areas where research and daily activities were synonymous. The rationale for the differing perception between departments was reported to be manifold and was often based around training; professional and disciplinary networks; and a cultural acceptance that research was integral to informing decisions. Literature points to some adult social care practitioners as having ‘fear’ of evidence and research– often perceived as being difficult and inaccessible [16]– this was exemplified by a study demonstrating that less than 2% of adult social care practitioners had knowledge and confidence in advanced data analysis skills and that only 10% had involvement in research in the past three years [23]. Commentators have pointed to a lack of confidence and capability in research in this particular staff base within local authorities, often attributed to only being ‘moderately’ equipped for research through their educational journey [24]. Conversely, the literature shows how public health teams in local authorities, as one example, are often viewed as holding a closer relationship with research than other departments [12, 15]. Literature shows how evidence usage is a specialism of public health professionals in local authorities and how the discipline of public health itself is situated in an evidence-focused arena [2, 7].

Terminology was an area where confusion existed across the departments. Some participants were unsure if they engaged in research, but did mention data gathering; intelligence; reflection; and other related terms. Such confusion has been reported in other local authorities [22] and indeed such methodological discourse about what constitutes research or otherwise can seem academic when pressures to deliver statutory services are pressing. It is, however, not surprising that such confusion exists as there are a plethora of definitions and views on research based on wide-ranging factors, such as: values; disciplinary bases; and institutional beliefs [25]. A research-view that embraces a myriad of approaches and methodologies seems pragmatic given the diverse schema of work that a local authority is responsible for. This would have to encompass a broad typology of evidence that can best answer the questions being asked within local communities– a ‘horses for courses’ approach, rather than a strict hierarchy of evidence [26].

The perceived level of engagement with external partners and organisations, particularly universities was another point of variance. Previous studies have identified trust between the local authority and university as a determining factor in whether these partnerships are robust [22]. This was not apparent in the data gathered in this study, rather relationships– even in research active departments– were suggested to be built on individual interests which could seem fragile and unsustainable. Previous studies have shown that links between local authorities and universities - especially local ones - are often based on individual arrangements. These connections tend to be stronger in professional fields where higher academic qualifications are required for practice [1, 16, 23]. The benefits of strong collaborative arrangements between local authorities and universities are apparent for not only generating and analysing data, but also for positive outcomes for communities [27]. Indeed the relationship is symbiotic with universities valuing the opportunities to demonstrate ‘real world’ impact through their scholarship [28].

Senior-level support was perceived to be crucial for fostering a strong research culture in local authorities. If senior leaders do not actively acknowledge and endorse research activities, staff may perceive that these efforts are not valued [22]. This study revealed that leaders rarely championed or explicitly valued space for ‘intellectual curiosity.’ However in instances where such curiosity was legitimised, research activities and engagement were positively perceived, fostering a more collaborative culture. Enabling intellectual curiosity within and across departmental areas—through ‘communities of practice’ involving local authority staff, academics and citizens—could help break down the siloed approaches currently present in this local authority [29] and move toward a more research-informed local authority typology [10]. Fynn et al. highlighted the importance of interdepartmental collaboration at Norfolk County Council (UK), noting that individuals with transferable research skills could contribute across services and departments. However, they also identified missed opportunities for sharing learning and research practices more widely throughout the Council [1]. Previous research has also shown the importance of ‘boundary-spanning’ leadership and cross-pollinating ideas and experiences in local authorities [1, 29, 30] and how these can be a driver for change [31]. Similar observations have also been seen in the healthcare sector where leadership has been influential for enhancing departmental research culture [14]. In the wider literature, calls have been made for more “organisational ambidexterity” pertaining to enabling the pursuit of intellectual curiosity *and* focusing on the day-to-day working pressures ([14], p355). The engagement of elected members with local authority leaders and staff also seems crucially important in relation to research leadership and while this study, and others [32], have shown good relationships between the local authority leaders and elected members, there were examples of more ad-hoc communication arrangements which may limit research evidence being translated in policy decisions. Research has suggested that observing evidence-informed decision-making by leaders and elected figures can enhance further engagement and uptake of research by staff [22]. While this feedback loop was not identified in this study, it would be worth exploring further, particularly through discussions with elected members and key decision-makers.

Overall, the study highlighted variability in capacity and capability for research in the organisation. Other research groups have developed a typology of research engagement from negligible activity (type 1) to comprehensive and systemic approaches (type 4) with one local authority identified as ‘type 2’– in effect, willing to engage in research with partners but not creating or using research independently [10]. The data here shows departmental areas working beyond this with evidence of research informed intervention development and leadership that is supportive. Of course, and has been demonstrated, there were departments more akin to ‘type 1’ in their capacity and capability for research highlighting the need for bespoke training and support for departments and personnel related to their research knowledge and experiences. A ‘whole system’ approach to research within the local authority– a concept put forward by Hock et al. [12] where interconnection and egalitarian relationships exist to address the complexity of research systems– was not evident in this study.

## Conclusions

Using evidence-informed policy can increase the likelihood of successful outcomes for citizens and communities [33]. However, local authorities do not always use evidence consistently in their decision-making processes and we know little about research capability and capacity in specific local authority departments [34]. This study focused on a single local authority in Northern England, focusing on the perceptions of 29 service managers and directors representing all directorates of the authority. This is the first time the views of these various constituents have been heard in relation to research capability and capacity. Data demonstrated variance between research practice; partnerships; and culture in departments and where space for intellectual curiosity was tempered by statutory obligations and service demands. There were exceptions to this, where departmental views of research were positive and leaders valued a research-informed culture. Prior training and educational backgrounds of staff was seen to be a key factor in research readiness and confidence; moreover, staff often working with acute needs and challenges found prioritisation of research a challenge. As echoed in the healthcare literature [14], research across departments in local authorities should be ‘championed’ and supported with it seen as ‘normal business’ acknowledging that research can benefit organisational change; policy-making; and outcomes for populations. Strong research culture values and integrates research as a routine part of practice, supported by both top-down leadership and bottom-up engagement through training, mentorship and support.

This research is, of course, a cross-sectional view in one local authority in England, but a body of work is growing which broadly supports the conclusions and findings. The study is unique in that it captures views from leaders across all areas of the local authority and demonstrates a more nuanced perspective of research capacity and capability. Such findings are also transferable to other international contexts where similar challenges in local authority research capability and capacity have been highlighted [2].

The findings should begin to inform HDRC areas who are funded to increase research capacity and capability across local authorities in England– in this local authority the HDRC is structurally located outside of the public health department and not led by this team as this could have potentially exacerbated wider capacity and capability inequalities within the organisation. There are clearly pockets of exemplary practice in the local authority and sharing this alongside training, support and development of staff will be essential. Further research is needed, with one useful area of investigation being to ascertain the views of elected members on evidence usage in their decision-making. This exploration of the ‘supply and demand’ for research is a critical factor in exploring research capability and capacity in local authorities [22].

## Data Availability

Data available on request from the corresponding author.
